# Early life exposures and school readiness: an observational cohort study using the Born in Bradford longitudinal birth cohort data

**DOI:** 10.1136/bmjpo-2025-003489

**Published:** 2026-01-04

**Authors:** Julia R Forman, Swapnil Ghotane, Ingrid Wolfe, Lorna Katharine Fraser

**Affiliations:** 1 Women and Children’s Health, King’s College London, London, UK; 2 Cicely Saunders Institute, King’s College London, London, UK

**Keywords:** Health Policy, Epidemiology, Child Health

## Abstract

**Objective:**

To assess the associations of early life exposures with school readiness.

**Design, setting, main outcome measure and participants:**

Using data from the Born in Bradford (BiB) birth cohort, we investigated the association of early life exposures and the likelihood of children achieving a ‘Good Level of Development’ (GLD) as measured by the Early Years Foundation Stage Profile at the end of their first school year. Multivariable logistic regression modelling was used to estimate associations of early life exposures with school readiness.

**Results:**

Outcome data were available for 10 589 of 13 858 BiB participants. 6272 (59%) children achieved a GLD. 17 variables were investigated, including child characteristics, maternal factors, family environment and socioeconomic exposures. In our multivariable model (n=7981), children with the following characteristics were significantly less likely to achieve a GLD: males (OR 0.43, 95% CI 0.39 to 0.47), Pakistani ethnicity (OR 0.76, 95% CI 0.66 to 0.89), younger children (OR 1.18 per month, 95% CI 1.16 to 1.19), preterm birth (gestational age<32 weeks OR 0.34, 95% CI 0.19 to 0.62; 32–36 weeks OR 0.64, 95% CI 0.51 to 0.80), younger maternal age (OR 1.01 per year of maternal age, 95% CI 1.00 to 1.02), lower maternal education (OR 0.27, 95% CI 0.20 to 0.37 with no qualifications, compared with higher degrees), never breastfed (OR 0.87, 95% CI 0.78 to 0.97), lower paternal employment status (OR 0.66, 95% CI 0.54 to 0.81, for unemployed, compared with non-manual employment), receiving benefits during pregnancy (OR 0.89, 95% CI 0.80 to 0.99) and eligible for free school meals (OR 0.71, 95% CI 0.62 to 0.81). A sensitivity analysis using multiple imputation (n=10 589) found consistent results.

**Conclusions:**

The exposures most strongly associated with low school readiness were male sex, low maternal education and preterm birth (<32 weeks gestation). Greater understanding of the exposures associated with school readiness can inform a proportionate universal approach to policymaking to improve school readiness and subsequent life outcomes.

**WHAT IS ALREADY KNOWN ON THIS TOPIC**

- Only 67.6% of children starting school in England in 2023 had achieved a Good Level of Development (GLD) at the end of their first year of schooling, as assessed by the Early Years Foundation Stage Profile (EYFSP).
- Achieving a GLD is associated with subsequent positive education and health outcomes.
- Many early life exposures and child characteristics are known to be associated with school readiness, including sex, socioeconomic status, age within the school year cohort, ethnicity, having English as an additional language and special educational needs.
- However, the adjusted associations of these exposures with the EYFSP, as assessed since 2013, have not been investigated.

**WHAT THIS STUDY ADDS**

- The EYFSP is a statutory assessment of school readiness for all children in England and is the policy-relevant outcome measure. This is the first study to estimate the adjusted associations of early life exposures with this measure of school readiness.
- 59% of children in the Born in Bradford cohort achieved a GLD compared with 65% nationally, during the 2013/2014–2015/2016 academic years.
- In the adjusted analysis, male sex, low levels of maternal education and birth before 32 weeks gestational age were the most strongly associated with low school readiness, while child characteristics, maternal factors, family environment and socioeconomic exposures were all significantly associated.

**HOW THIS STUDY MIGHT AFFECT RESEARCH, PRACTICE OR POLICY**

- Many of the exposures shown in this work to have important associations with school readiness are routinely collected or available in administrative data sets.
- Therefore, it may be possible to enhance identification of children or populations at greatest risk of not being ready for school using routinely collected data and better target provision of the necessary support.
- In addition, greater understanding of modifiable exposures associated with school readiness may improve school readiness by informing policies that influence these exposures.

## Introduction

 Children begin formal education with differences in their development, skills and learning.^1^ School readiness is associated with educational success and later life outcomes,^2^,^7^ and early interventions deliver the greatest economic returns on investment.^8^ As such, there is policy interest in supporting children to be prepared for formal education. In England, approximately two-thirds of children are ready for school, and this has changed very little in recent years.^1^ The current government has set a milestone to improve school readiness to 75% by 2028.^9^

Interventions in the early years, often focused on physical, cognitive, behavioural and social and emotional development, can support child development and school readiness.^10 11^ Universal services such as health visiting, and other early years services and education settings, aim to support children, identify and address development and learning needs, and promote school readiness. In England, universal services have been under pressure due to funding shortfalls and severe workforce shortages, particularly in health visiting.^12 13^ In light of this, increased targeting of services may be advisable, but identifying the children most likely to benefit remains a challenge. With a better understanding of the exposures associated with school readiness, it may be possible to use routinely collected data to enhance identification of children at greatest risk of not being ready for school and offer appropriate support.

School readiness can be measured in several ways. In England, the Early Years Foundation Stage Profile (EYFSP) is a statutory assessment of all children’s development conducted at the end of the academic year in which children turn five. The Good Level of Development (GLD) measure from the EYFSP is the most widely used measure of school readiness in the UK. In the most recently reported academic year 2023/2024, 67.7% of students assessed in England achieved a GLD. Girls were more likely than boys to achieve a GLD (74.1% vs 60.6%), and disparities were also reported for children with English as an additional language (EAL), children eligible for free school meals, children from minority ethnic groups and children born later in the academic year who therefore are among the youngest within their cohort.^1^

Exposures during early childhood, a critical period for development, impact children’s readiness for formal education.^14^ Analysis of prenatal and early childhood exposures and school readiness, as measured by the EYFSP, has been hindered by the lack of availability of linked education data. The Born in Bradford (BiB) birth cohort includes linked education data, including the EYFSP, and detailed information on early life exposures.^15 16^ The aim of this study was to estimate the associations of early life exposures and school readiness, as measured by the EYFSP, using data from the BiB birth cohort.

## Methods

### Overview

Using the BiB longitudinal birth cohort data set, we used univariable and multivariable analyses to investigate the impact of early life exposures on the likelihood of children achieving a GLD, as assessed by the EYFSP.

### Setting and participants

The BiB data set (n=13 858 children born between 2007 and 2011) contains demographic and clinical data, and linked education records from the National Pupil Database, which provides data on education outcomes, including the EYFSP. The EYFSP was revised in 2013 and is not directly comparable with pre-2013 EYFSP; therefore, we only included participants with recorded assessments of the post-2013 version of the EYFSP.

### Outcome variable

Children in state-funded, early-years education in England are assessed by their teachers using the EYFSP during the final term of the academic year in which they turn 5 years old. Children are assessed to have achieved a GLD (binary outcome) in the EYFSP if they are at or exceeding the expected level for all 12 Early Learnings Goals within five areas: communication and language; personal, social and emotional development; physical development; literacy; and mathematics.^17^

### Exposure variables

**Figure 1:**
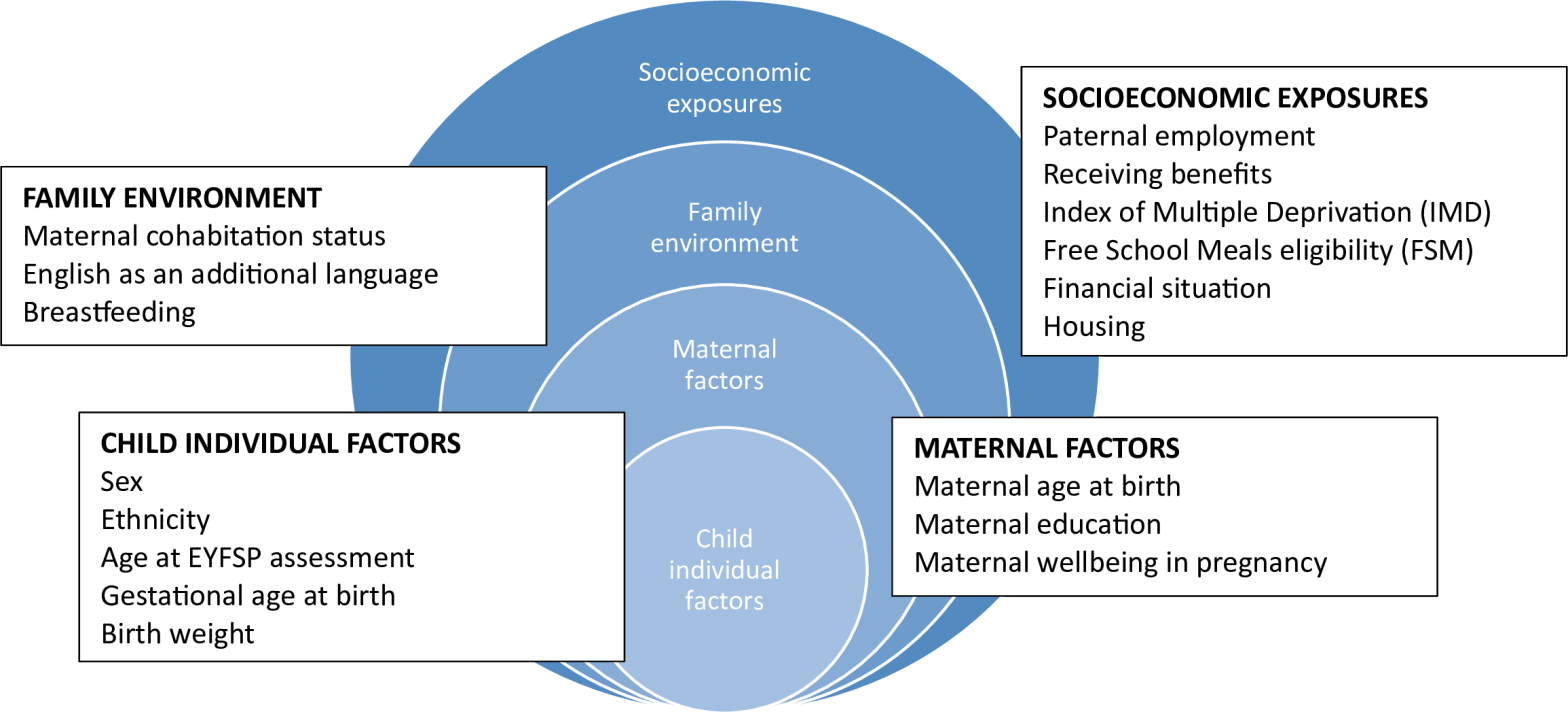
Grouping of exposure variables. Socio-ecological model describing the exposures analysed, grouped as child individual factors, maternal factors, family environment and socioeconomic factors. EYFSP, Early Years Foundation Stage Profile.

Variables were identified as potential exposures with important associations with school readiness from the published literature. 17 of these variables were available or could be derived from data within the BiB data set, with <50% missingness. The exposure variables are grouped using an adapted socio-ecological model: child individual factors, maternal factors, family environment and socioeconomic factors (figure 1). The following text and online supplemental table 1 describe these variables and how they were derived or categorised.

The five ‘child individual factors’ are sex (female and male), ethnicity (grouped as white, mixed, Indian, Pakistani, Bangladeshi, Black and other), estimated age at EYFSP assessment (months), gestational age at birth (categorised as extremely or very preterm if less than 32 weeks, moderate to late preterm if 32 to 36 weeks, full term if 37 weeks or later) and birth weight (coded as low if <2500 g, normal/high if ≥2500 g).

The three ‘maternal factors’ are maternal age at child’s birth (years), maternal education (nine levels, treated as categories, see details in online supplemental table 1) and maternal well-being assessed in pregnancy at 26–28 weeks gestation using the General Health Questionnaire.

The three ‘family environment factors’ are cohabitation status during pregnancy (married and living with partner; not married and living with partner or not living with partner), EAL (yes or no) and breastfeeding status which was coded as ‘breastfed’ if any breastfeeding was recorded, and otherwise ‘no recorded breastfeeding’. Data on EAL are collected annually as part of the education data; here, we used the data from year 5 as this had a low level of missingness (1%), while there were high levels of missing data in reception (99%) and earlier years.

Six measures of socioeconomic status were investigated. Paternal employment status during pregnancy is described by six categories: employed-non-manual, employed-manual, self-employed, student, unemployed and don’t know. Receiving means-tested benefits during pregnancy is categorised yes or no. The Index of Multiple Deprivation (IMD) categories were created within the BiB data set using the IMD quintiles within Bradford as cut-offs to create five groups. Children in receipt of free school meals are categorised as yes or no. (Due to high levels of missing data in reception year, year 1 data were used.) Maternally reported financial situation in pregnancy was recorded with six categories: living comfortably; doing alright; just about getting by; quite difficult; very difficult and does not wish to answer. Housing tenure during pregnancy was coded into seven categories: owns outright; mortgage; rent free; private landlord; social housing; other and don’t know.

### Data analysis

Analyses were conducted using Stata 18.0.^18^ Descriptive statistics are reported for each exposure, by the GLD outcome, to describe the prevalence of each exposure in the sample, and in the full BiB cohort. Univariable logistic regression analyses were performed to calculate ORs and 95% CIs to assess the unadjusted association of each variable with GLD.

Multivariable logistic regression modelling was used to describe adjusted associations between exposures and the GLD outcome; ORs and 95% CIs are reported. We included 11 of the 17 candidate exposures in our multivariable model. Birth weight and EAL were excluded due to strong associations with other exposures (gestational age and ethnicity, respectively; see online supplemental tables 2 and 3), and maternal well-being due to its weak association with the outcome in the univariable analysis. Due to the richness of the data set, we had six candidate measures for socioeconomic status, and we included three (paternal employment status during pregnancy; benefits during pregnancy; free school meal eligibility). We prioritised developing a model that could use data in early life to identify children at highest risk of not being school ready, and therefore we excluded data that are not routinely collected before starting school, that is, the parental assessment of financial situation and housing type. (Maternal well-being also is not routinely measured in pregnancy.) IMD category was excluded as it describes area-level rather than familial socioeconomic status, while the other measures of socioeconomic status are family-specific.

Missing data are summarised in online supplemental table 4. Complete cases were used for the primary analysis for transparency and simplicity. Multiple imputation using chained equations was used as a sensitivity analysis.^19^ The outcome and the 11 exposures in our multivariable model were included in the imputation; ten imputation data sets were produced using ‘mi impute chained’ in Stata, before analysis and pooling with the ‘mi estimate’ command.

### Patient and public involvement

BiB conducts patient and public involvement (PPI), including with parent governors and young ambassadors. No additional PPI was conducted for this study.

## Results

### Participants

**Figure 2:**
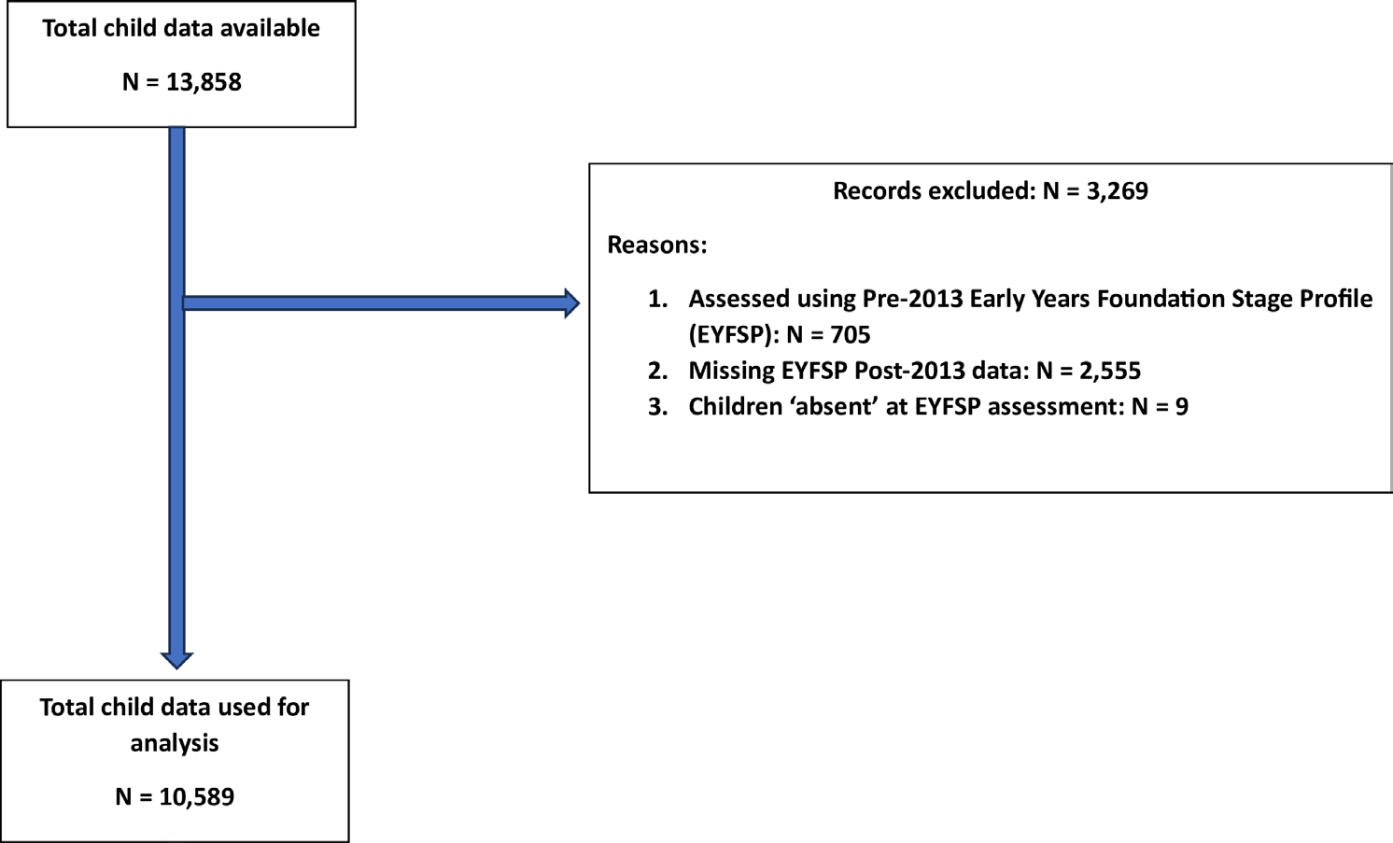
Total Born in Bradford participant data used for analysis.

Data from the BiB longitudinal birth cohort^15 16^ were provided on 13 858 children born between 2007 and 2011 (figure 2). From the initial 13 858 participants, data from 10 589 participants were included in our analyses (online supplemental table 4). 3269 participants were excluded as data were not available for the post-2013 EYFSP assessment. Of these, 705 participants were assessed using the pre-2013 EYFSP, 2555 are missing EYFSP assessment data, and 9 were absent at the EYFSP assessment. The characteristics of the included cohort are similar to those of the full BiB cohort, though proportions of missingness differ (online supplemental table 4).

### Exposure variables

In a review of the literature, 19 variables in the BiB data were identified as potential exposures with important associations with school readiness.^20^,^30^ 17 of these variables were available or could be derived from data within the BiB data set with <50% missingness (online supplemental table 1). Two variables were excluded from our analyses due to high levels of missingness in the BiB data: maternal smoking (75% missing) and alcohol consumption during pregnancy (75% missing). Other variables of potential interest that were not recorded in the BiB data include breastfeeding duration, nursery attendance, other measures of pollution or environmental exposures, child development measures, parental attachment measures and whether parent(s) spent time in care as a child.

### Analysis findings

6272 (59%) children achieved a GLD (online supplemental table 4). There were low levels of missing data (<3%) for child characteristics, maternal age at child’s birth, EAL and free school meals status; and moderate missingness (15%–29%) for maternal education, all socioeconomic exposures except free school meals, cohabitation status and maternal well-being.

Online supplemental table 5 presents the unadjusted and adjusted ORs. In univariable analyses, children with the following characteristics were significantly less likely to have achieved a GLD: males (OR 0.49, 95% CI 0.45 to 0.53) compared with females, Pakistani ethnicity (OR 0.81, 95% CI 0.75 to 0.89) compared with white ethnicity, younger children (OR 1.16, increase per month of child age, 95% CI 1.14 to 1.17), born preterm (both categories; see online supplemental table 5) compared with children born at term, low birth weight (OR 0.63, 95% CI 0.55 to 0.72) compared with normal birth weight, younger maternal age (OR 1.02, increase per year of maternal age, 95% CI 1.01 to 1.03), lower maternal education (all categories below degree level; see online supplemental table 5), mother not living with partner (OR 0.73, 95% CI 0.64 to 0.81) compared with mother married and living with partner, having EAL (OR 0.70, 95% CI 0.64 to 0.75), no recorded breastfeeding (OR 0.79, 95% CI 0.73 to 0.86) compared with any recorded breastfeeding and lower socioeconomic status by all measures (see online supplemental table 5). Maternal well-being was not found to be significantly associated with the outcome.

**Figure 3:**
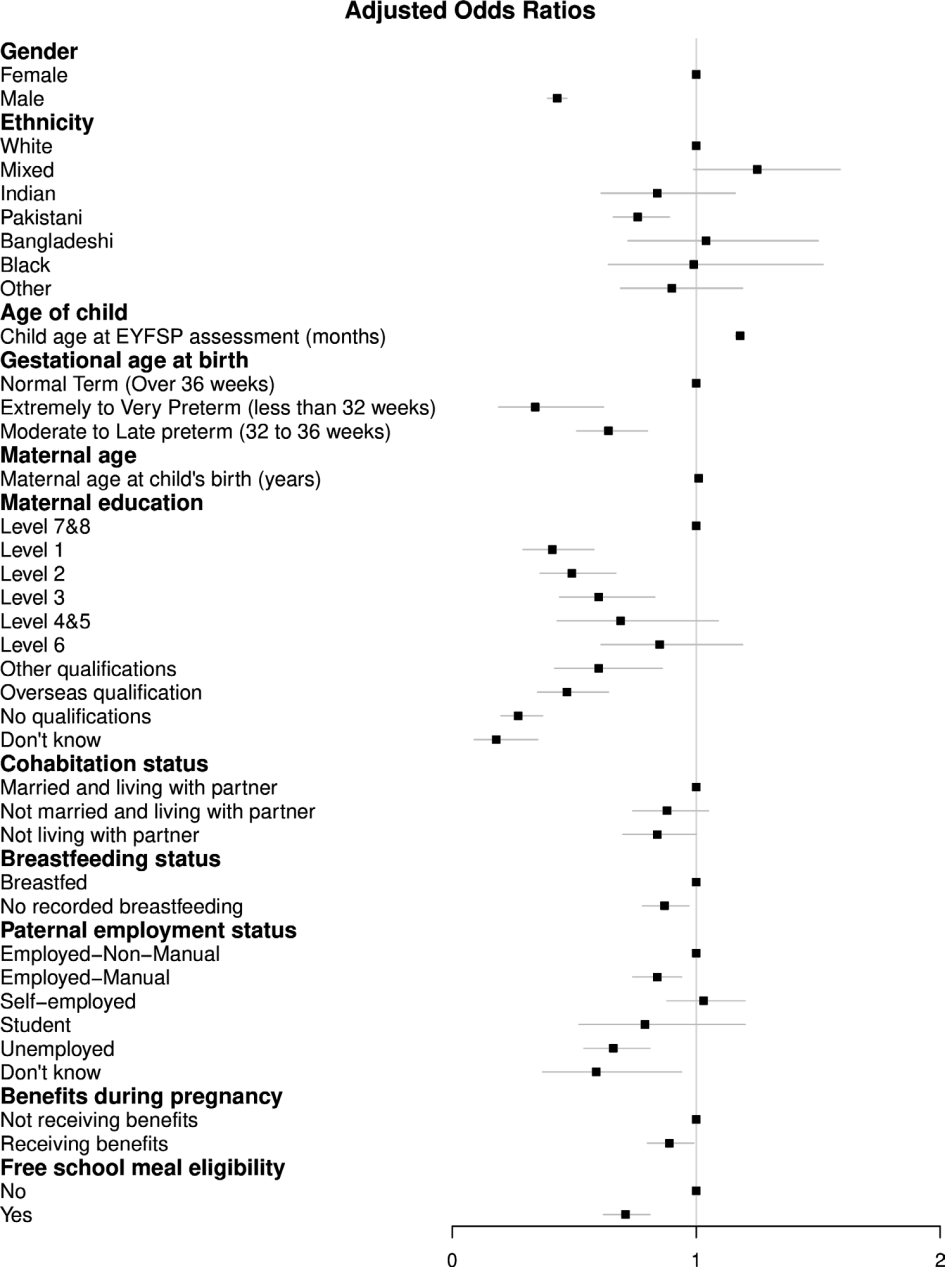
A forest plot of the adjusted odds ratios of attaining a Good Level of Development, estimated using logistic regression. EYFSP, Early Years Foundation Stage Profile.

In our multivariable model (online supplemental table 5 and figure 3), with 7981 complete cases, children with the following characteristics were significantly less likely to have achieved a GLD: males (OR 0.43, 95% CI 0.39 to 0.47) compared with females, Pakistani ethnicity (OR 0.76, 95% CI 0.66 to 0.89) compared with white ethnicity, younger children (OR 1.18 per month, 95% CI 1.16 to 1.19), preterm birth (gestational age<32 weeks OR 0.34, 95% CI 0.19 to 0.62; 32–36 weeks OR 0.64, 95% CI 0.51 to 0.80) compared with normal term, younger maternal age (OR 1.01 per year of maternal age, 95% CI 1.00 to 1.02), lower maternal education (see online supplemental table 5), never breastfed (OR 0.87, 95% CI 0.78 to 0.97) compared with ever breastfed, father unemployed (OR 0.66, 95% CI 0.54 to 0.81), in manual employment (OR 0.84, 95% CI 0.74 to 0.94) or of unknown employment status (OR 0.59, 95% CI 0.37 to 0.94) compared with father in non-manual employment, receiving benefits during pregnancy (OR 0.89, 95% CI 0.80 to 0.99) compared with not receiving benefits in pregnancy and receiving free school meals (OR 0.71, 95% CI 0.62 to 0.81) compared with not receiving free school meals.

In our multivariable model with multiple imputation, we included 10 589 participants. The results are very similar, with two minor differences. First, in the multiple imputation analysis, there is a significant association between the outcome and mixed ethnicity (OR 1.27, 95% CI 1.04 to 1.54) compared with white ethnicity, while in the complete cases analysis the only significant association with ethnicity was in the Pakistani ethnic group. Second, the association between breastfeeding status and the outcome is not significant in the multiple imputation analysis (OR 0.91, 95% CI 0.83 to 1.00). In both cases, the estimated ORs are similar to those estimated using complete cases.

## Discussion

### Findings

Children in the BiB birth cohort, assessed during the 2013/2014–2015/2016 academic years, were found to have low levels of school readiness; 59% achieved a GLD compared with 65% nationally.^1^ Children of Pakistani ethnicity, the largest ethnic group, were significantly less likely to achieve a GLD compared with white children. 76% of children in this group have EAL (see online supplemental tables), and EAL was significantly associated with the outcome in univariable analyses. As EAL was not included in the multivariable model, due to its relationship with ethnicity in this cohort, further work in other populations will be needed to assess its impact. While this study is the first to describe adjusted associations using the EYFSP as the outcome measure, the associations with other exposures were similar to previous work. We note that the strongest associations in our adjusted model are with male sex, preterm birth<32 weeks gestational age and with low or overseas maternal education (figure 3). Importantly, boys constitute 51% of the population, and children of mothers with education at or below level 2 comprise 38% of the study population, with an additional 16% having overseas educational qualifications.

### Strengths and limitations

The BiB birth cohort provides a rich data set, including identification of mother–father–child triads and linkages to education and primary care data, which enabled this novel analysis of the EYFSP outcome, the current policy-relevant measure routinely collected for all children in England. Furthermore, the exposures investigated are routinely collected by universal services in early life, which will facilitate development of a predictive model that can be employed in service provision and targeting.

As the BiB data represent a regional data set, these findings may lack external validity and generalisability to national or international populations. While the data set is rich, there may be other important exposures that we were not able to include, for example, alcohol and tobacco use. Furthermore, due to variability in breastfeeding recording, breastfeeding status was dichotomised (‘breastfed’ if any breastfeeding was recorded, and otherwise ‘no recorded breastfeeding’). Although no association was found between maternal well-being assessed in pregnancy and school readiness, other work suggests that the association of school readiness and postpartum maternal well-being may deserve further consideration.^31^ Due to recruitment to the cohort at 26 weeks gestation, the cohort is disproportionately missing participants born in the extreme preterm group. Missing outcome data may result from failure to link study participants to education data or to missing EYFSP data in the education data. Common among longitudinal studies, withdrawal from study participation or other loss-to-follow-up generates missing data. A sensitivity analysis using multiple imputation confirmed that results were consistent with the complete cases analysis. In addition, self-reported and subjectively assessed data may introduce bias. Finally, the associations identified in this work may not be causal.

### Comparison with previous studies

The findings presented here corroborate previous work investigating school readiness in the UK and internationally, and its associations with sex, ethnicity, socioeconomic status and maternal age, education and mental health measures. Previous studies used a range of school readiness assessments, some of which were routinely collected while others were research specific. None of these studies used the post-2013 version of the EYFSP.^20^,^30^ Most recently, Bandyopadhyay *et al* identified significant associations between school readiness and sex, age, gestational age at birth, birth weight, maternal age, breastfeeding and socioeconomic factors using linked routinely collected Welsh data. Their model did not include ethnicity or maternal education, which were found to be significant exposures in this study.^20^ Camacho *et al* previously developed a predictive risk model for school readiness, using the UK Millennium Cohort Study (MCS). Their study showed significant associations between school readiness and sex, ethnicity, maternal education, breastfeeding, housing and socioeconomic factors (social class and annual income). In contrast with the work presented here, maternal age was not significantly associated with school readiness, and child’s age, gestational age at birth and parental cohabitation were not included in the predictive risk model.^21^ Among the exposures that were common between these previous studies and our work, we see good agreement between the estimated adjusted ORs, with some differences in ethnicity and maternal education in the Camacho work. We hypothesise that the greater association with ethnicity observed in the Camacho work may be due to differences in the cohorts; the MCS is nationally representative, while BiB is regional. Our findings also align with research from low-income and middle-income countries (LMICs), where there are marked inequalities in early experiences and outcomes.^32^ Jeong *et al* report that the top five factors associated with co-occurrence of stunting and off-track early childhood development in LMICs were low socioeconomic status, no early childhood education, low maternal education, no toys at home and diarrhoea.^33^ Our work adds to the previous literature by providing insights into the adjusted associations with the EYFSP assessment, the outcome measure currently in routine use across England, including adjusted associations for exposures that are routinely collected, identifying significant associations for all except parental cohabitation.

### Policy implications

School readiness has important implications for educational success and later life outcomes, and perpetuates inequalities, and is therefore of public health importance^2^,^4^ and a current policy focus.^9^ The findings of this study may shape policy in two ways.

First, through understanding the associations between exposures and school readiness, and using routinely collected linked data, policies, investment and supportive interventions can be targeted to reach those least likely to be school ready. This targeting can be introduced at the individual level, through development and implementation of predictive risk models, or at the local population level. Where unmet needs are identified, evidence-informed policies and services will be required. There is evidence for speech, language and communication interventions and for social, emotional and cognitive development.^34^ However, implementation may be challenging due to existing funding and workforce shortages in this sector. Where existing universal services are not able to reach the entire population, data-enhanced targeting may facilitate adoption of a proportionate universal approach, focusing existing resources where there is greatest need.

Secondly, policymakers can consider which exposures are modifiable and causal (causality is not assessed in this work), and seek to improve population school readiness through policies that influence these exposures. Preconception and perinatal care and policies can prevent unwanted pregnancies in young women,^35^ reduce preterm births through primary and secondary prevention,^36^ and influence breastfeeding choices.^37^ The strong associations between socioeconomic status and school readiness expose the potential for policies on child poverty. Policies on age of school entry, and flexibility to defer school entry, may impact school readiness. Other exposures may be indirectly influenced or moderated through policy. For example, maternal education may be modifiable or low maternal education may be moderated through support to enrich the child’s learning environments, at home and in community settings.^38^

## Conclusions

Child characteristics, maternal factors, family environment and socioeconomic exposures were all significantly associated with school readiness in this first study to estimate the adjusted associations of early life exposures and school readiness using the post-2013 EYFSP. Common exposures strongly associated with low school readiness are male sex and low levels of maternal education. Birth before 32 weeks gestational age is also strongly associated with low school readiness. These findings may be used to shape policy and target interventions and resources to individuals or populations, in a proportionate universal approach, in order to improve early child development and subsequent life outcomes, and reduce inequalities.

## Supplementary material

10.1136/bmjpo-2025-003489 online supplemental file 1

## Data Availability

Data may be obtained from a third party and are not publicly available.
