# Supplementary material for: Early life exposures and school readiness: an observational cohort study using the Born in Bradford longitudinal birth cohort data
Source: BMJ Paediatr Open. 2026 Jan 4;10(1):e003489. doi: 10.1136/bmjpo-2025-003489 (PMC12778278; doi:10.1136/bmjpo-2025-003489)
Supplement: online supplemental file 1 [file bmjpo-10-1-s001.docx]

# Early life exposures and school readiness: an observational cohort study using the Born in Bradford longitudinal birth cohort data - Supplementary Materials

## Supplementary Table 1. Exposures and variable details including derivation and handling

| **Exposure** | **Available in Bradford (BiB) dataset** | **Born in Bradford (BiB) variable details** | | | **Variable categories, derivation, and handling** |
| --- | --- | --- | --- | --- | --- |
| **Child Individual Factors** |  | ***Table ID*** | ***Variable*** | ***Label*** |  |
| 1. Sex | Yes | BiB_CohortInfo.child_info | admincgender | Child gender | 1. Male 2. Female |
| 1. Ethnicity | Yes | BiB_CohortInfo.ethnicity | ethnicity16 | Ethnicity - 16 categories  1 : English, Welsh, Scottish, Northern Irish or British  2 : Irish  3 : Any other White background  4 : White and Black Caribbean  5 : White and Black African  6 : White and Asian  7 : Any other Mixed background  8 : Indian  9 : Pakistani  10 : Bangladeshi  11 : Chinese  12 : Any other Asian background  13 : African  14 : Caribbean  15 : Other Black, African or Caribbean background  16 : Any other ethnic group | 16 categories merged into 7 categories:   1. White (1-3 of 16) 2. Mixed (4-7 of 16) 3. Indian 4. Pakistani 5. Bangladeshi 6. Black (13-15 of 16) 7. Other (11, 12, and 16) |
| 1. Child age information    1. Age at EYFSP assessment    2. Month of birth    3. Academic term (season) of birth | 1. Yes 2. Yes 3. Derived | 1. BiB_Education_Record.edrecs_eyfsp_2 2. BiB_CohortInfo.person_info 3. BiB_CohortInfo.person_info | 1. age_months_est 2. MonthOfBirth 3. Derived (Seasonal) | 1. Estimated age in months at assessment 2. Participant month of birth 3. Seasonal term of birth | 1. Treated as a continuous variable 2. N/A 3. Derived from month of birth into 3 categories as: 4. Autumn (Sept to Dec) 5. Spring (Jan to Apr) 6. Summer (May to Aug) |
| 1. Gestational age at birth | Derived | BiB_Pregnancy.eclipse_preg | eclgestwks | Gestation to last completed week | Categorised as:   1. Extremely Preterm (less than 28 weeks) 2. Very Preterm (28 to less than 32 weeks) 3. Moderate to Late preterm (32 to 36 weeks) 4. Normal Term (37 weeks and later) |
| 1. Birth weight (<2500 grams) | Derived | BiB_Pregnancy.eclipse_baby | eclbirthwt | Birth weight (grams) | Categorised as:   1. Low (<2500 grams) 2. Normal/High (≥2500 grams) |
| **Maternal factors** |  |  |  |  |  |
| 1. Maternal age at child’s birth | Yes | BiB_CohortInfo.child_info | admincdobagemy | Mother age at child date of birth (years) | N/A |
| 1. Maternal education | Yes | BiB_Baseline.base_m_survey | edu0mumeuk | Mum’s highest education qualification – 14 categories:  1 : 1 + 0 levels/CSEs/GCEs(any grades)  2 : 5 + 0 levels, 5+ CSEs (grade 1) 5 + GCSEs, School Certificate  3 : 1 + A levels/AS levels  4 : 2 + A levels, 4 + AS levels, Higher School certificate  5 : NVQ Level 1, Foundation GNVQ  6 : NVQ Level 2,Intermediate GNVQ  7 : NVQ Level 3, Advanced GNVQ  8 : NVQ Levels 4-5, HNC, HND  9 : First Degree (e.g. BA, BSc)  10 : Higher Degree (e.g. MA, PhD, PGCE Post-grad cert/diploma)  11 : Other qualifications (e.g. City and Guilds, RSA/OCR, BTEC)  12 : Overseas qualification  13 : No Qualifications  14 : Don’t know | 14 categories merged into 9 categories to match 9 levels of UK qualification as:   1. Level 1   [NVQ Level 1, Foundation GNVQ & 1 + 0 levels/CSEs/GCEs(any grades)]   1. Level 2   [NVQ Level 2, Intermediate GNVQ & 5 + 0 levels, 5+ CSEs (grade 1) 5 + GCSEs, School Certificate]   1. Level 3   [1 + A levels/AS levels & 2 + A levels, 4 + AS levels, Higher School certificate & NVQ Level 3, Advanced GNVQ]   1. Level 4&5   [NVQ Levels 4-5, HNC, HND]   1. Level 6   [First Degree (e.g. BA, BSc)]   1. Level 7&8   [Higher Degree (e.g. MA, PhD, PGCE Post-grad cert/diploma)]  Other qualifications (e.g. City and Guilds, RSA/OCR, BTEC)   1. Overseas qualification 2. No qualifications 3. Don't know |
| 1. Maternal wellbeing during pregnancy | Yes | BiB_Baseline.base_m_survey | ghq0totalscore | GHQ-28 total score | Treated as a continuous variable |
| **Family environment** |  |  |  |  |  |
| 1. Cohabitation status | Yes | BiB_Baseline.base_m_survey | hhd0marchb | Marital and cohabitation status combined (derived) | Categories are:   1. Married and living with partner 2. Not married and living with partner   Not living with partner |
| 1. Breastfeeding status | Derived | Primary Care data: ChildHealth_Breastfeeding | ctv3term | Feeding information of child from birth | If any breastfeeding recorded:   1. Breastfed   If no records of breastfeeding:  No recorded breastfeeding |
| 1. English as an additional language (recorded in Year 5) | Yes | BiB_Education_Record.edrecs_annual_context | edcont_eal | Does child have English as a second language? | Note: Used data from School Year 5 due to low completion in earlier years. (99% missing in Reception, 85% in Year 1.). Categories are:   1. Yes   No |
| **Socioeconomic factors** |  | ***Table ID*** | ***Variable*** | ***Label*** |  |
| 1. Paternal employment status / Socioeconomic class | Yes | BiB_Baseline.base_m_survey | job0fthemp | Father’s employment status | 6 categories are as follows:   1. Employed-Non-Manual 2. Employed-Manual 3. Self-employed 4. Student 5. Unemployed 6. Don’t know |
| 1. Receiving benefits | Yes | BiB_Baseline.base_m_survey | ben0mentst | Means tested benefits received | 1. Yes 2. No |
| 1. Free school meal eligibility at Year 1 | Yes | BiB_Education_Record.edrecs_annual_context | edcont_fsm | Is child in receipt of free school meals? | Note: Year 1 chosen as high level of missing data for Reception year. Categories are:   1. Yes 2. No |
| 1. Index of Multiple Deprivation (IMD) | Yes | BiB_Baseline.base_m_survey | imdquintileswithinbradford | IMD Quintiles (within Bradford) | N/A |
| 1. Financial situation | Yes | BiB_Baseline.base_m_survey | fin0manfin | How well mother and husband/partner managing financially | Categories are:  1. Living comfortably 2. Doing alright  3. Just about getting by 4. Quite difficult 5. Very difficult 6. Does not wish to answer |
| 1. Type of housing | Yes | BiB_Baseline.base_m_survey | res0hseten | Housing tenure (derived) | Categories are:   1. Owns outright 2. Mortgage 3. Rent free 4. Private landlord 5. Social housing 6. Other 7. Don’t know |

## Supplementary Table 2. Number of participants with English as an Additional Language, by ethnicity

|  | **English as an Additional Language** | | | |
| --- | --- | --- | --- | --- |
| Ethnicity | *No* | *Yes* | *Missing* | % EAL |
| White | 3622 | 218 | 66 | 6% |
| Bangladeshi | 37 | 293 | 2 | 88% |
| Black | 115 | 42 | 3 | 26% |
| Indian | 98 | 182 | 9 | 63% |
| Mixed | 533 | 58 | 6 | 10% |
| Other | 159 | 267 | 3 | 62% |
| Pakistani | 1133 | 3657 | 84 | 75% |
| Missing | 0 | 1 | 1 | 50% |

## Supplementary Table 3. Number of participants per birth weight category, by gestational age

|  | **Birth weight** | |  |  |
| --- | --- | --- | --- | --- |
| **Gestational age** | *Normal or high* | *Low* | | *Missing* |
| Normal term | 9320 | 478 | | 0 |
| Moderate to late preterm | 199 | 345 | | 1 |
| Extremely or very preterm | 1 | 80 | | 165 |

## Supplementary Table 4. Description of family and child characteristics by school readiness (attaining a GLD on the EYFSP) for BiB participants with complete post-2013 EYFSP GLD data (study sample) compared with the full BiB cohort participants

|  | Exposures | | Study Sample | | | | | | Full Cohort | |
| --- | --- | --- | --- | --- | --- | --- | --- | --- | --- | --- |
|  |  |  | School ready | | Not school ready | | Total | | Total | |
|  |  |  | N | % (row) | N | % (row) | N | % (column) | N | % (column) |
|  | Total | | 6272 | 59 | 4317 | 41 | 10589 |  | 13858 |  |
| Child Individual Factors | | |  |  |  |  |  |  |  |  |
| 1 | **Sex** | Male | 2756 | *51* | 2646 | *49* | 5402 | *51* | 7153 | *52* |
|  |  | Female | 3516 | *68* | 1671 | *32* | 5187 | *49* | 6704 | *48* |
|  |  | **Missing** | 0 | *0* | 0 | *0* | 0 | *0* | 1 | *0.01* |
| 2 | **Ethnicity** | White | 2407 | *62* | 1499 | *38* | 3906 | *37* | 5300 | *38* |
|  |  | Mixed | 373 | *62* | 224 | *38* | 597 | *6* | 737 | *5* |
|  |  | Indian | 185 | *64* | 104 | *36* | 289 | *3* | 444 | *3* |
|  |  | Pakistani | 2767 | *57* | 2107 | *43* | 4874 | *46* | 6001 | *43* |
|  |  | Bangladeshi | 194 | *58* | 138 | *42* | 332 | *3* | 395 | *3* |
|  |  | Black | 98 | *61* | 62 | *39* | 160 | *2* | 270 | *2* |
|  |  | Other | 247 | *58* | 182 | *42* | 429 | *4* | 600 | *4* |
|  |  | **Missing** | 1 | *0.01* | 1 | *0.01* | 2 | *0.02* | 111 | *1* |
| 3 | **Age at EYFSP assessment (months)** | 56 | 150 | *38* | 248 | *62* | 398 | *4* | 399 | *3* |
|  |  | 57 | 298 | *39* | 466 | *61* | 764 | *7* | 764 | *6* |
|  |  | 58 | 342 | *44* | 432 | *56* | 774 | *7* | 774 | *6* |
|  |  | 59 | 342 | *46* | 405 | *54* | 745 | *7* | 747 | *5* |
|  |  | 60 | 366 | *48* | 395 | *52* | 761 | *7* | 762 | *5* |
|  |  | 61 | 472 | *57* | 351 | *43* | 823 | *8* | 824 | *6* |
|  |  | 62 | 522 | *61* | 339 | *39* | 861 | *8* | 862 | *6* |
|  |  | 63 | 612 | *61* | 389 | *39* | 1001 | *9* | 1001 | *7* |
|  |  | 64 | 646 | *64* | 367 | *36* | 1013 | *10* | 1014 | *7* |
|  |  | 65 | 671 | *68* | 313 | *32* | 984 | *9* | 984 | *7* |
|  |  | 66 | 719 | *74* | 246 | *25* | 965 | *9* | 967 | *7* |
|  |  | 67 | 755 | *75* | 256 | *25* | 1011 | *10* | 1011 | *7* |
|  |  | 68 plus | 377 | *77* | 112 | *23* | 489 | *5* | 489 | *4* |
|  |  | **Missing** | 0 | *0* | 0 | *0* | 0 | *0* | 3260 | *24* |
| 4 | **Gestational age at birth** | Extremely to Very Preterm (less than 32 weeks) | 30 | *37* | 51 | *63* | 81 | *1* | 150 | *1* |
|  |  | Moderate to Late preterm (32 to 36 weeks) | 270 | *50* | 275 | *50* | 545 | *5* | 756 | *6* |
|  |  | Normal Term (Over 36 weeks) | 5880 | *60* | 3918 | *40* | 9789 | *93* | 12620 | *91* |
|  |  | **Missing** | 92 | *56* | 73 | *44* | 165 | *2* | 332 | *2* |
| 5 | **Birthweight** | Low (less than 2500gm) | 443 | *49* | 460 | *51* | 903 | *9* | 1260 | *9* |
|  |  | Normal/High | 5736 | *60* | 3784 | *40* | 9520 | *90* | 12264 | *89* |
|  |  | **Missing** | 93 | *56* | 73 | *44* | 166 | *2* | 334 | *2* |
| Maternal factors | | |  |  |  |  |  |  |  |  |
| 6 | **Maternal age at child’s birth in Years** | Under 20 | 555 | *52* | 507 | *48* | 1062 | *10* | 1427 | *10* |
|  |  | 21 to 30 | 3772 | *59* | 2658 | *41* | 6430 | *61* | 8374 | *61* |
|  |  | 31 to 40 | 1851 | *63* | 1102 | *37* | 2953 | *28* | 3878 | *28* |
|  |  | Above 40 | 94 | *65* | 50 | *35* | 144 | *1* | 179 | *1* |
|  |  | **Missing** | 0 | *0* | 0 | *0* | 0 | *0* | 0 | *0* |
| 7 | **Maternal education** | Level 1 [NVQ Level 1, Foundation GNVQ & 1 + 0 Levels/Cses/Gces(Any Grades)] | 314 | *56* | 251 | *44* | 565 | *5* | 704 | *5* |
|  |  | Level 2 [NVQ Level 2,Intermediate GNVQ & 5 + 0 Levels, 5+ Cses (Grade 1) 5 + Gcses, School Certificate | 1283 | *60* | 838 | *40* | 2121 | *20* | 2702 | *20* |
|  |  | Level 3 [1 + A Levels/AS Levels & 2 + A Levels, 4 + AS Levels, Higher School Certificate & NVQ Level 3, Advanced GNVQ] | 784 | *66* | 399 | *33* | 1183 | *11* | 1485 | *11* |
|  |  | Level 4&5 [NVQ Levels 4-5, HNC, HND] | 118 | *71* | 48 | *29* | 166 | *2* | 233 | *2* |
|  |  | Level 6 [First Degree (E.G. BA, Bsc)] | 567 | *76* | 183 | *24* | 750 | *7* | 1079 | *8* |
|  |  | Level 7&8 [Higher Degree (E.G. MA, Phd, PGCE Post-Grad Cert/Diploma)] | 283 | *79* | 73 | *21* | 356 | *3* | 552 | *4* |
|  |  | Other Qualifications (E.G. City And Guilds, RSA/OCR, BTEC) | 302 | *68* | 141 | *32* | 443 | *4* | 631 | *5* |
|  |  | Overseas Qualification | 996 | *60* | 672 | *40* | 1668 | *16* | 2215 | *16* |
|  |  | No Qualifications | 619 | *45* | 767 | *55* | 1386 | *13* | 1766 | *13* |
|  |  | Don't Know | 25 | *37* | 42 | *63* | 67 | *1* | 81 | *1* |
|  |  | **Missing** | 981 | *52* | 903 | *48* | 1884 | *18* | 2410 | *17* |
| 8 | **Maternal wellbeing in pregnancy** | GHQ total score recorded | 4578 | *61* | 2968 | *39* | 7546 | *71* | 9582 | *69* |
|  |  | GHQ total score mean (95% CI) | 22.9 (22.6 – 23.2) | | 23.3 (22.9 – 23.7) | | 23.1 (22.8 -23.3) | | 23.2 (23.0 – 23.4) | |
|  |  | **Missing** | 1694 | *56* | 1349 | *44* | 3043 | *29* | 4267 | *31* |
| Family environment | | |  |  |  |  |  |  |  |  |
| 9 | **Cohabitation status** | Married And Living With Partner | 3563 | *62* | 2201 | *38* | 5764 | *54* | 7533 | *54* |
|  |  | Not Married And Living With Partner | 943 | *63* | 546 | *37* | 1489 | *14* | 2032 | *15* |
|  |  | Not Living With Partner | 784 | *54* | 666 | *46* | 1450 | *14* | 1884 | *14* |
|  |  | **Missing** | 982 | *52* | 904 | *48* | 1886 | *18* | 2409 | *17* |
| 10 | **English as an additional language** | Yes | 2580 | *55* | 2138 | *45* | 4718 | *45* | 5183 | *37* |
|  |  | No | 3601 | *63* | 2096 | *37* | 5697 | *54* | 6334 | *46* |
|  |  | **Missing** | 91 | *52* | 83 | *48* | 174 | *1* | 2341 | *17* |
| 11 | **Breastfeeding status** | Breastfed | 3489 | *62* | 2155 | *38* | 5644 | *53* | 7308 | *53* |
|  |  | No recorded breastfeeding | 2697 | *56* | 2089 | *44* | 4786 | *45* | 5891 | *42* |
|  |  | **Missing** | 86 | *54* | 73 | *46* | 159 | *2* | 659 | *5* |
| Socioeconomic factors | |  |  |  |  |  |  |  |  |  |
| 12 | **Paternal employment status during pregnancy** | Employed-Non-Manual | 2190 | *67* | 1077 | *33* | 3267 | *31* | 4403 | *32* |
|  |  | Employed-Manual | 1670 | *57* | 1267 | *43* | 2937 | *28* | 3729 | *27* |
|  |  | Self-employed | 790 | *64* | 447 | *36* | 1237 | *12* | 1645 | *12* |
|  |  | Student | 58 | *54* | 50 | *46* | 108 | *1* | 185 | *1* |
|  |  | Unemployed | 315 | *47* | 351 | *53* | 666 | *6* | 865 | *6* |
|  |  | Don't Know | 41 | *42* | 57 | *58* | 98 | *1* | 142 | *1* |
|  |  | **Missing** | 1208 | *53* | 1068 | *47* | 2276 | *21* | 2889 | *21* |
| 13 | **Benefits status during pregnancy** | Yes | 2089 | *56* | 1664 | *44* | 3753 | *35* | 4692 | *34* |
|  |  | No | 3202 | *65* | 1748 | *35* | 4950 | *47* | 6743 | *49* |
|  |  | **Missing** | 981 | *52* | 905 | *48* | 1886 | *18* | 2423 | *17* |
| 14 | **IMD category** | 1 (most deprived) | 1847 | *57* | 1402 | *43* | 3249 | *31* | 4213 | *30* |
|  |  | 2 | 1370 | *58* | 1011 | *42* | 2381 | *22* | 3117 | *22* |
|  |  | 3 | 1073 | *65* | 574 | *35* | 1647 | *16* | 2138 | *15* |
|  |  | 4 | 773 | *70* | 339 | *30* | 1112 | *10* | 1458 | *11* |
|  |  | 5 | 219 | *72* | 85 | *28* | 304 | *3* | 392 | *3* |
|  |  | **Missing** | 990 | *52* | 906 | *48* | 1896 | *18* | 2540 | *18* |
| 15 | **Free school meal eligibility at Year 1** | Yes | 1123 | *48* | 1208 | *52* | 2331 | *22* | 2557 | *19* |
|  |  | No | 5099 | *62* | 3086 | *38* | 8185 | *77* | 9067 | *65* |
|  |  | **Missing** | 50 | *68* | 23 | *32* | 73 | *1* | 2234 | *16* |
| 16 | **Financial situation** | Living Comfortably | 1435 | *65* | 778 | *35* | 2213 | *21* | 3041 | *22* |
|  |  | Doing Alright | 2213 | *60* | 1446 | *40* | 3659 | *35* | 4744 | *34* |
|  |  | Just About Getting By | 1287 | *59* | 886 | *41* | 2173 | *21* | 2744 | *20* |
|  |  | Quite Difficult | 265 | *55* | 217 | *45* | 482 | *5* | 655 | *5* |
|  |  | Very Difficult | 77 | *51* | 74 | *49* | 151 | *1* | 216 | *2* |
|  |  | Does Not Wish To Answer | 13 | *43* | 17 | *57* | 30 | *0.28* | 40 | *0.29* |
|  |  | **Missing** | 982 | *52* | 899 | *48* | 1881 | *18* | 2418 | *17* |
| 17 | **Type of housing** | Owns outright | 727 | *58* | 520 | *42* | 1247 | *12* | 1607 | *12* |
|  |  | Mortgage | 2630 | *65* | 1425 | *35* | 4055 | *38* | 5225 | *38* |
|  |  | Rent free | 438 | *61* | 275 | *39* | 713 | *7* | 857 | *6* |
|  |  | Private landlord | 928 | *60* | 630 | *40* | 1558 | *15* | 2226 | *16* |
|  |  | Social Housing | 474 | *50* | 465 | *50* | 939 | *9* | 1260 | *9* |
|  |  | Other | 47 | *47* | 52 | *53* | 99 | *1* | 145 | *1* |
|  |  | Don’t Know | 51 | *51* | 49 | *49* | 100 | *1* | 131 | *1* |
|  |  | **Missing** | 977 | *52* | 901 | *48* | 1878 | *18* | 2407 | *17* |

## Supplementary Table 5. Unadjusted (all variables) and adjusted (11 variables) logistic regression associations for the exposures with attaining a Good Level of Development on the EYFSP (complete cases and imputed)

|  | **Dependent variable: Good Learning Development (GLD):** 0 = Not school ready 1 = School ready | **Unadjusted** | | | | |  | **Adjusted, complete cases**  **(n = 7,981)** | | |  | **Adjusted, imputed**  **(n = 10,589)** | | |
| --- | --- | --- | --- | --- | --- | --- | --- | --- | --- | --- | --- | --- | --- | --- |
|  |  | **No. of**  **observations**  **(N)** |  | **OR** | **95% conf. interval** | **p value** |  | **OR** | **95% conf. interval** | **p value** |  | **OR** | **95% conf. interval** | **p value** |
| **Child Individual Factors** | | |  |  |  |  |  |  |  |  |  |  |  |  |
| 1 | Sex | **10,589** | **Female** | 1 |  |  |  | 1 |  |  |  | 1 |  |  |
|  |  |  | Male | 0.49 | 0.45 - 0.53 | **0.001** |  | 0.43 | 0.39 - 0.47 | **0.001** |  | 0.45 | 0.41 - 0.49 | **0.001** |
|  |  |  |  |  |  |  |  |  |  |  |  |  |  |  |
| 2 | Ethnicity | **10,587** | **White** | 1 |  |  |  | 1 |  |  |  | 1 |  |  |
|  |  |  | Mixed | 1.03 | 0.86 - 1.23 | 0.68 |  | 1.25 | 0.99 - 1.59 | 0.062 |  | 1.27 | 1.04 - 1.54 | **0.019** |
|  |  |  | Indian | 1.10 | 0.86 - 1.42 | 0.42 |  | 0.84 | 0.61 - 1.16 | 0.298 |  | 0.92 | 0.69 - 1.23 | 0.570 |
|  |  |  | Pakistani | 0.81 | 0.75 - 0.89 | **0.001** |  | 0.76 | 0.66 - 0.89 | **0.001** |  | 0.83 | 0.72 - 0.95 | **0.006** |
|  |  |  | Bangladeshi | 0.87 | 0.69 - 1.09 | 0.25 |  | 1.04 | 0.72 - 1.50 | 0.825 |  | 0.88 | 0.67 - 1.16 | 0.370 |
|  |  |  | Black | 0.98 | 0.71 - 1.36 | 0.92 |  | 0.99 | 0.64 - 1.52 | 0.960 |  | 1.01 | 0.70 - 1.45 | 0.974 |
|  |  |  | Other | 0.84 | 0.69 - 1.03 | 0.100 |  | 0.90 | 0.69 - 1.19 | 0.475 |  | 0.85 | 0.67 - 1.07 | 0.172 |
|  |  |  |  |  |  |  |  |  |  |  |  |  |  |  |
| 3 | Age of child at EYFSP assessment, in months | **10,589** | **Age as continuous variable** | 1 |  |  |  |  |  |  |  |  |  |  |
|  |  |  | Mean age 62.37 (range 56-72) | 1.16 | 1.14 - 1.17 | **0.001** |  | 1.18 | 1.16 - 1.19 | **0.001** |  | 1.18 | 1.16 - 1.19 | **0.001** |
|  |  |  |  |  |  |  |  |  |  |  |  |  |  |  |
| 4 | Gestational age at birth | **10,424** | **Normal Term (Over 36 weeks)** | 1 |  |  |  |  |  |  |  |  |  |  |
|  |  |  | Extremely to Very Preterm (less than 32 weeks) | 0.39 | 0.24 - 0.61 | **0.001** |  | 0.34 | 0.19 - 0.62 | **0.001** |  | 0.34 | 0.21 - 0.56 | **0.001** |
|  |  |  | Moderate to Late preterm (32 to 36 weeks) | 0.65 | 0.54 - 0.77 | **0.001** |  | 0.64 | 0.51 - 0.80 | **0.001** |  | 0.62 | 0.51 - 0.75 | **0.001** |
|  |  |  |  |  |  |  |  |  |  |  |  |  |  |  |
| 5 | Birth weight | **10,423** | **Normal/High** | 1 |  |  |  |  |  |  |  |  |  |  |
|  |  |  | Low (less than 2500gm) | 0.63 | 0.55 - 0.72 | **0.001** |  |  |  |  |  |  |  |  |
|  |  |  |  |  |  |  |  |  |  |  |  |  |  |  |
| **Maternal factors** | | |  |  |  |  |  |  |  |  |  |  |  |  |
| 6 | Maternal age at child’s birth (years) | **10,598** | **Age as continuous variable** | **1.02** | 1.01 - 1.03 | **0.001** |  | 1.01 | 1.00 - 1.02 | **0.020** |  | 1.01 | 1.00 - 1.02 | **0.006** |
|  |  |  | Mean age 27.5 (range 15-49) |  |  |  |  |  |  |  |  |  |  |  |
|  |  |  |  |  |  |  |  |  |  |  |  |  |  |  |
| 7 | Maternal education | **8,705** | **Level 7&8 [Higher Degree (e.g. MA, PhD, PGCE Post-grad cert/diploma)]** | 1 |  |  |  | 1 |  |  |  | 1 |  |  |
|  |  |  | Level 1 [NVQ Level 1, Foundation GNVQ & 1 + 0 levels/CSEs/GCEs(any grades)] | 0.32 | 0.23 - 0.43 | **0.001** |  | 0.41 | 0.29 - 0.58 | **0.001** |  | 0.41 | 0.29 - 0.57 | **0.001** |
|  |  |  | Level 2 [NVQ Level 2, Intermediate GNVQ & 5 + 0 levels, 5+ CSEs (grade 1) 5 + GCSEs, School Certificate | 0.39 | 0.30 - 0.51 | **0.001** |  | 0.49 | 0.36 - 0.67 | **0.001** |  | 0.50 | 0.37 - 0.68 | **0.001** |
|  |  |  | Level 3 [1 + A levels/AS levels & 2 + A levels, 4 + AS levels, Higher School certificate & NVQ Level 3, Advanced GNVQ] | 0.50 | 0.38 - 0.67 | **0.001** |  | 0.6 | 0.44 - 0.83 | **0.002** |  | 0.62 | 0.44 - 0.86 | **0.005** |
|  |  |  | Level 4&5 [NVQ Levels 4-5, HNC, HND] | 0.63 | 0.41 -0.96 | **0.04** |  | 0.69 | 0.43 - 1.09 | 0.111 |  | 0.67 | 0.42 - 1.07 | 0.093 |
|  |  |  | Level 6 [First Degree (e.g. BA, BSc)] | 0.79 | 0.58 - 1.08 | 0.15 |  | 0.85 | 0.61 - 1.19 | 0.347 |  | 0.89 | 0.65 - 1.22 | 0.471 |
|  |  |  | Other qualifications (e.g. City and Guilds, RSA/OCR, BTEC) | 0.55 | 0.39 - 0.76 | **0.001** |  | 0.60 | 0.42 - 0.86 | **0.005** |  | 0.61 | 0.43 - 0.86 | **0.004** |
|  |  |  | Overseas qualification | 0.38 | 0.29 - 0.50 | **0.001** |  | 0.47 | 0.35 - 0.64 | **0.001** |  | 0.47 | 0.35 - 0.64 | **0.001** |
|  |  |  | No qualifications | 0.20 | 0.15 - 0.27 | **0.001** |  | 0.27 | 0.2 - 0.37 | **0.001** |  | 0.27 | 0.20 - 0.36 | **0.001** |
|  |  |  | Don't know | 0.15 | 0.08 - 0.26 | **0.001** |  | 0.18 | 0.09 - 0.35 | **0.001** |  | 0.20 | 0.10 - 0.38 | **0.001** |
|  |  |  |  |  |  |  |  |  |  |  |  |  |  |  |
| 8 | Maternal wellbeing during pregnancy | **7,546** | Total GHQ Score | 0.99 | 0.99-1.00 | 0.10 |  |  |  |  |  |  |  |  |
|  |  |  | Mean GHQ score 23.25 (range 0-78) |  |  |  |  |  |  |  |  |  |  |  |
|  |  |  |  |  |  |  |  |  |  |  |  |  |  |  |
| **Family environment** | | |  |  |  |  |  |  |  |  |  |  |  |  |
| 9 | Cohabitation status | **8,703** | **Married and living with partner** | 1 |  |  |  | 1 |  |  |  | 1 |  |  |
|  |  |  | Not married and living with partner | 1.06 | 0.94 - 1.20 | 0.28 |  | 0.88 | 0.74 - 1.05 | 0.156 |  | 0.89 | 0.75 - 1.05 | 0.152 |
|  |  |  | Not living with partner | 0.73 | 0.64 - 0.81 | **0.001** |  | 0.84 | 0.70 - 1.00 | 0.052 |  | 0.87 | 0.73 - 1.04 | 0.127 |
|  |  |  |  |  |  |  |  |  |  |  |  |  |  |  |
| 10 | English as an additional language | **10,415** | **No** | 1 |  |  |  |  |  |  |  |  |  |  |
|  |  |  | Yes | 0.7 | 0.64 - 0.75 | **0.001** |  |  |  |  |  |  |  |  |
|  |  |  |  |  |  |  |  |  |  |  |  |  |  |  |
| 11 | Breastfeeding status | **10,423** | **Breastfed** | 1 |  |  |  |  |  |  |  |  |  |  |
|  |  |  | No recorded breastfeeding | 0.79 | 0.73 - 0.86 | **0.001** |  | 0.87 | 0.78 - 0.97 | **0.011** |  | 0.91 | 0.83 – 1.00 | 0.054 |
|  |  |  |  |  |  |  |  |  |  |  |  |  |  |  |
| **Socioeconomic factors** | | |  |  |  |  |  |  |  |  |  |  |  |  |
| 12 | Paternal employment status  during pregnancy | **8,313** | **Employed-Non-Manual** | 1 |  |  |  | 1 |  |  |  | 1 |  |  |
|  |  |  | Employed-Manual | 0.65 | 0.59 - 0.72 | **0.001** |  | 0.84 | 0.74 - 0.94 | **0.003** |  | 0.81 | 0.72 - 0.92 | **0.001** |
|  |  |  | Self-employed | 0.86 | 0.75 - 0.99 | **0.040** |  | 1.03 | 0.88 - 1.20 | 0.697 |  | 0.99 | 0.85 - 1.15 | 0.898 |
|  |  |  | Student | 0.57 | 0.39 - 0.84 | **0.004** |  | 0.79 | 0.52 - 1.20 | 0.270 |  | 0.72 | 0.46 - 1.11 | 0.135 |
|  |  |  | Unemployed | 0.44 | 0.37 - 0.52 | **0.001** |  | 0.66 | 0.54 - 0.81 | **0.001** |  | 0.66 | 0.53 - 0.81 | **0.001** |
|  |  |  | Don't know | 0.35 | 0.23 - 0.53 | **0.001** |  | 0.59 | 0.37 - 0.94 | **0.026** |  | 0.57 | 0.33 - 0.99 | **0.045** |
|  |  |  |  |  |  |  |  |  |  |  |  |  |  |  |
| 13 | Benefits during pregnancy | **8,703** | **Not receiving benefits** | 1 |  |  |  | 1 |  |  |  | 1 |  |  |
|  |  |  | Receiving benefits | 0.68 | 0.62 - 0.74 | **0.001** |  | 0.89 | 0.80 - 0.99 | **0.028** |  | 0.89 | 0.80 - 0.99 | **0.028** |
|  |  |  |  |  |  |  |  |  |  |  |  |  |  |  |
| 14 | Free school meal eligibility (recorded at Year 1) | **10,516** | **No** | 1 |  |  |  | 1 |  |  |  | 1 |  |  |
|  |  |  | Yes | 0.56 | 0.51 - 0.61 | **0.001** |  | 0.71 | 0.62 – 0.81 | **0.001** |  | 0.75 | 0.67 - 0.84 | **0.001** |
|  |  |  |  |  |  |  |  |  |  |  |  |  |  |  |
| 15 | IMD category | **8,693** | **5 (least deprived)** | 1 |  |  |  |  |  |  |  |  |  |  |
|  |  |  | 1 | 0.51 | 0.39 – 0.66 | **0.001** |  |  |  |  |  |  |  |  |
|  |  |  | 2 | 0.52 | 0.40 – 0.68 | **0.001** |  |  |  |  |  |  |  |  |
|  |  |  | 3 | 0.72 | 0.55 – 0.95 | **0.02** |  |  |  |  |  |  |  |  |
|  |  |  | 4 | 0.88 | 0.66 – 1.17 | 0.39 |  |  |  |  |  |  |  |  |
|  |  |  |  |  |  |  |  |  |  |  |  |  |  |  |
| 16 | Financial situation during pregnancy | **8,708** | **Living comfortably** | 1 |  |  |  |  |  |  |  |  |  |  |
|  |  |  | Doing alright | 0.82 | 0.74 - 0.92 | **0.001** |  |  |  |  |  |  |  |  |
|  |  |  | Just about getting by | 0.78 | 0.69 - 0.88 | **0.001** |  |  |  |  |  |  |  |  |
|  |  |  | Quite difficult | 0.66 | 0.54 - 0.80 | **0.001** |  |  |  |  |  |  |  |  |
|  |  |  | Very difficult | 0.56 | 0.40 - 0.78 | **0.001** |  |  |  |  |  |  |  |  |
|  |  |  | Does not wish to answer | 0.41 | 0.20 - 0.85 | **0.02** |  |  |  |  |  |  |  |  |
|  |  |  |  |  |  |  |  |  |  |  |  |  |  |  |
| 17 | Type of housing  during pregnancy | **8,711** | **Mortgage** | 1 |  |  |  |  |  |  |  |  |  |  |
|  |  |  | Owns outright | 0.75 | 0.66 - 0.85 | **0.001** |  |  |  |  |  |  |  |  |
|  |  |  | Rent free | 0.86 | 0.73 - 1.01 | 0.080 |  |  |  |  |  |  |  |  |
|  |  |  | Private landlord | 0.79 | 0.70 - 0.89 | **0.001** |  |  |  |  |  |  |  |  |
|  |  |  | Social housing | 0.55 | 0.47 - 0.63 | **0.001** |  |  |  |  |  |  |  |  |
|  |  |  | Other | 0.49 | 0.32 - 0.73 | **0.001** |  |  |  |  |  |  |  |  |
|  |  |  | Don’t know | 0.56 | 0.37 - 0.83 | **0.005** |  |  |  |  |  |  |  |  |
