# Supplementary material for: Early life exposures and school readiness: an observational cohort study using the Born in Bradford longitudinal birth cohort data
Source: BMJ Paediatr Open. 2026 Jan 4;10(1):e003489. doi: 10.1136/bmjpo-2025-003489 (PMC12778278; doi:10.1136/bmjpo-2025-003489)
Supplement: Supplementary file 2 [file bmjpo-2025-003489.reviewer_comments.pdf]

PEER REVIEW HISTORY

BMJ Paediatrics Open publishes all reviews undertaken for accepted manuscripts. Reviewers are asked to complete a checklist review form and are provided with free text boxes to elaborate on their assessment. These free text comments are reproduced below.

ARTICLE DETAILS

**TITLE (PROVISIONAL)**	Early life exposures and school readiness: an observational cohort study using the Born in Bradford longitudinal birth cohort data
**AUTHORS**	Forman, Julia; Ghotane, Swapnil; Wolfe, Ingrid; Fraser, Lorna

VERSION 1 - REVIEW

**REVIEWER NAME**	Peter Flom
**REVIEWER AFFILIATION**	Peter Flom Consulting
**REVIEWER CONFLICT OF INTEREST**	N/A
**AI DISCLOSURE**	No
**DATE REVIEW RETURNED**	20-Mar-2025

**GENERAL COMMENTS**	I confine my remarks to statistical and methodological aspects of this paper. The general approach is fine, but I do have some points that should be addressed before I can recommend publication.<br><br>The authors should be careful of causal language such as "impact" (in the abstract). "Association" is better.<br><br>In general, I do not like dichotomous measures of variables such as child development. They are less powerful than analysis using continuous measures, and, clearly, child development is not dichotomous. Here, there is a statutory issue that makes it a little more defensible to use it. But, if the EYFSP provides scores as well as a dichotomy, the authors might also do either an OLS or ordinal regression, or perhaps some kind of count regression of the number of goals. \I won't insist on this, but it would be nice to have and may provide additional insight.<br><br>A pet peeve of mine is using "gender" when you mean "sex". "Male" and :"Female" are sexes (and there are other possibilities, but they are rare). "Masculine" and "feminine" are genders (and it's more like several continua). Maybe BMP Peds has a style sheet on this, I'm just pointing it out.<br><br>On p. 7 please say more about how you excluded certain measures. There are other ways of dealing with colinearity such as principal component regression, factor analysis before the regression, or ridge regression. Were those considered? I'm not saying what you did was incorrect, but it would be nice to have a discussion (maybe in an online supplement). But I wouldn't insist on that.<br><br>Using MICE as a sensitivity analysis is fine, although I'm not sure MICE results are any more confusing that the complete case results..

	How was the maternal education variable treated? Ordinal variables as IVs are tricky. There are some fancy methods, but treating them as categorical can be OK, just say what was done.<br><br>This is a style issue, but shouldn't the numbers in the tables be flush right?<br><br>Finally, at least on my screen, the tables were hard to read and were bigger than the screen. That will probably be fixed in production.<br><br>Despite all these comments, I think the authors did a good job.<br><br>Peter Flom

VERSION 1 – AUTHOR RESPONSE

	Reviewer: 1 Dr. Peter Flom, Peter Flom Consulting	
	I confine my remarks to statistical and methodological aspects of this paper. The general approach is fine, but I do have some points that should be addressed before I can recommend publication.	Thank you for your thoughtful feedback.
	The authors should be careful of causal language such as "impact" (in the abstract). "Association" is better.	We have replaced “impact” with “association” in the Abstract, and in the Key Messages, Introduction, and Conclusions.
	In general, I do not like dichotomous measures of variables such as child development. They are less powerful than analysis using continuous measures, and, clearly, child development is not dichotomous. Here, there is a statutory issue that makes it a little more defensible to use it. But, if the EYFSP provides scores as well as a dichotomy, the authors might also do either an OLS or ordinal regression, or perhaps some kind of count regression of the number of goals. I won't insist on this, but it would be nice to have and may provide additional insight.	We share your concerns regarding dichotomized measures. Our decision to use the “Good Level of Development” in this instance was due to its use in UK government policy, as you note.
	A pet peeve of mine is using "gender" when you mean "sex". "Male" and :"Female" are sexes (and there are other possibilities, but they are rare). "Masculine" and "feminine" are genders (and it's more like several continua). Maybe BMP Peds has a style sheet on this, I'm just pointing it out.	We have replaced “gender” with “sex”.
	On p. 7 please say more about how you excluded certain measures. There are other	We did not employ regression methods beyond those described in the manuscript.

	ways of dealing with colinearity such as principal component regression, factor analysis before the regression, or ridge regression. Were those considered? I'm not saying what you did was incorrect, but it would be nice to have a discussion (maybe in an online supplement). But I wouldn't insist on that.	We prioritized developing a model that could use data in early life to identify children at highest risk of not being school ready, and therefore we excluded data that is not routinely collected before starting school, i.e. the parental assessment of financial situation and housing type. IMD category was also excluded as it describes area-level rather than familial socioeconomic status, while the included measures of socioeconomic status are family-specific (Paternal employment status during pregnancy; Benefits during pregnancy; Free school meal eligibility).<br><br>Birth weight and English as an additional language were excluded due to strong associations with gestational age and ethnicity, respectively; these associations are illustrated in the Supplementary Tables. Maternal wellbeing was excluded due to its weak association with the outcome in the univariable analysis.<br><br>To summarize, of our seventeen candidate exposures, we excluded the six described above in our final model.<br><br>We have edited the text to clarify this point (page 7).
	Using MICE as a sensitivity analysis is fine, although I'm not sure MICE results are any more confusing that the complete case results.	We chose to present the complete case results as the primary analysis for transparency and simplicity, and to compare these with the MICE results as a sensitivity analysis. We recognize that alternatively we could have presented the MICE results and compared them with the complete case results.
	How was the maternal education variable treated? Ordinal variables as IVs are tricky. There are some fancy methods, but treating them as categorical can be OK, just say what was done.	Maternal education was treated as a categorical variable. We have edited the text to clarify this on page 6:

		“maternal education (9 levels, treated as categories…)”
	This is a style issue, but shouldn't the numbers in the tables be flush right?<br><br>Finally, at least on my screen, the tables were hard to read and were bigger than the screen. That will probably be fixed in production.	We will work with the production team to improve readability.
	Despite all these comments, I think the authors did a good job.	Thank you for your constructive comments and kind remarks.
